# Rapid Initiation of Hyperbaric Oxygen Therapy for Multiple Simultaneous Cases of Acute Carbon Monoxide Poisoning at a Single Center

**DOI:** 10.1093/milmed/usaf100

**Published:** 2025-04-03

**Authors:** Takayuki Kurokawa, Ichiyo Ohara, Chie Watanabe, Koji Kuwata, Itsumi Hashimoto, Manabu Kitagaki, Takehiko Murakami

**Affiliations:** Department of Clinical Service, Japan Self-Defense Forces Hospital Yokosuka, Yokosuka, Kanagawa 237-0071, Japan; Department of Defense Medicine, National Defense Medical College, Saitama 359-8513, Japan; Department of Clinical Service, Japan Self-Defense Forces Hospital Yokosuka, Yokosuka, Kanagawa 237-0071, Japan; Department of Clinical Service, Japan Self-Defense Forces Hospital Yokosuka, Yokosuka, Kanagawa 237-0071, Japan; Department of Internal Medicine, National Defense Medical College, Saitama 359-8513, Japan; Department of Clinical Service, Japan Self-Defense Forces Hospital Yokosuka, Yokosuka, Kanagawa 237-0071, Japan; Department of Internal Medicine, National Defense Medical College, Saitama 359-8513, Japan; Department of Clinical Service, Japan Self-Defense Forces Hospital Yokosuka, Yokosuka, Kanagawa 237-0071, Japan; Department of Preventive Medicine and Public Health, National Defense Medical College, Saitama 359-8513, Japan; Department of Clinical Service, Japan Self-Defense Forces Hospital Yokosuka, Yokosuka, Kanagawa 237-0071, Japan; Bureau of Personnel and Education, Japan Ministry of Defense, Shinjuku, Tokyo 162-8801, Japan; Department of Clinical Service, Japan Self-Defense Forces Hospital Yokosuka, Yokosuka, Kanagawa 237-0071, Japan; Medical Science Course, School of Clinical Sciences, Kanagawa Dental University, Yokosuka, Kanagawa 236-8580, Japan

## Abstract

**Introduction:**

Hyperbaric oxygen therapy (HBOT) is used to treat acute carbon monoxide poisoning. However, few reports have detailed its use in large patient cohorts, and optimal management guidelines have yet to be established. Herein, we report the clinical presentation and simultaneous treatment of multiple patients experiencing acute carbon monoxide poisoning on an offshore ship within the territorial waters of Japan.

**Case presentation:**

Eleven patients were promptly transported to our hospital from a Japanese Maritime Self-Defense Force destroyer owing to accidental acute carbon monoxide poisoning. We opted to treat this incident as a mass casualty and immediately appointed a medical control officer and established medical teams. The medical control officer guided the general treatment plan and coordinated with the various sections, and the medical teams treated the patients. The patients were immediately administered normobaric oxygen via a facial mask. Those with the most severe conditions underwent simultaneous HBOT within 3 hours of hospital arrival. Two and 4 weeks after the second therapy session, all symptoms had resolved, with no physical or cognitive sequelae.

**Conclusion:**

We report the successful treatment of patients with concurrent acute carbon monoxide poisoning via HBOT at a single facility. This report highlights the feasibility of efficacious treatment at a single facility in scenarios in which multiple individuals experience carbon monoxide poisoning. It is important that all staff members, including those in administration, understand the concept of disaster medicine. Additionally, in HBOT facilities, regular training is needed for events involving a large number of HBOT-indicated patients.

## INTRODUCTION

Carbon monoxide (CO) is generated by the incomplete combustion of organic matter. In confined spaces, CO accumulation can result in acute CO poisoning (ACOP). In accidental events, mass exposure of a large number of individuals to CO may occur simultaneously.^1^

Our 100-bed hospital is managed by the Japanese Maritime Self-Defense Force (JMSDF). It is equipped with 2 type-2 multiplace hyperbaric oxygen therapy (HBOT) chambers and actively performs HBOT. A similar type-2 HBOT facility near our hospital is managed by another JMSDF squadron.

In this report, we describe the simultaneous treatment of multiple patients afflicted with ACOP on an offshore ship within the territorial waters of Japan. Notably, initial HBOT was immediately administered, and no neurological sequelae were encountered.

## CASE PRESENTATION

Ethical standards were applied in this human research study.

In September 2017, a civilian worker (patient A in **Table 1**) on a JMSDF destroyer experienced malaise and loss of consciousness while performing repairs inside a sewage tank with a running engine generator. His colleagues attempted to rescue him but subsequently experienced similar symptoms (**Table 1**). Although ventilation within the tank took place indoors, the exhaust gas from the generator was released outdoors through a duct.

**Table 1.** Patient characteristics

							Blood CO-Hb (%)			
Pt	Age (years),sex	SDF/Civ	LOC	Symptoms	Medical history	Smoker	Pre-HBOT	Day 0	Day 1	HBOT protocol
A	65, M	Civ	Yes	Numbness, fatigue, headache	None	Yes	41.5	2.0	0.8	TT6 × 3, TT5 × 2
B	73, M	Civ	No	Eye field abnormality, headache	None	Yes	37.4	3.6	0.8	TT6 × 3, TT5 × 2
C	67, M	Civ	No	None	None	Yes	36.1	2.7	0.9	TT6 × 3, TT5 × 2
D	40, M	Civ	No	Numbness, fatigue	Type 2 DM	Yes	31.8	0.8	0.0	TT6 × 3, TT5 × 2
E	38, F	Civ	Yes	Numbness, hyperventilation	None	Yes	18.6	0.8	0.5	TT6 × 3, TT5 × 2
F	49, M	SDF	No	Dull headache	None	Yes	22.8	0.6	0.6	TT6 × 2, TT5 × 3
G	23, M	SDF	No	Dull headache	None	Yes	15.8	0.5	0.5	TT6 × 1, TT5 × 2
H	29, M	SDF	No	Dull headache	None	Yes	10.1	0.6	0.5	TT6 × 1, TT5 × 2
I	46, M	SDF	No	Chills	None	Yes	8.7	0.8	0.7	TT6 × 1, TT5 × 2
J	46, M	SDF	No	Dull headache	None	No	6.4	0.6	0.6	TT6 × 1, TT5 × 2
K	29, M	SDF	No	Dull headache	None	Yes	3.9	0.3	0.3	TT6 × 1, TT5 × 2
L	27, M	SDF	No	Headache, nausea	None	Yes	3.5	–	0.8	TT6 × 1, TT5 × 2
M	46, M	SDF	No	Headache	None	Yes	2.8	–	0.5	TT6 × 2, TT5 × 1
N	39, M	SDF	No	Headache	None	Yes	1.0 (Day 1)	–	0.4	TT5 × 3
O	47, M	SDF	No	Eye field abnormality, headache	None	Yes	1.4 (Day 1)	–	0.9	TT5 × 3
P	51, M	SDF	No	None	None	Yes	3.6 (Day 1)	–	1.1	TT5 × 3

Abbreviations: Pt, patient; M, male; F, female; SDF, Self-Defense Force personnel; Civ, civilian; LOC, loss of consciousness; HBOT, hyperbaric oxygen therapy; CO-Hb, carboxyhemoglobin; TT, treatment table; DM, diabetes mellitus.

**Figure 1.**
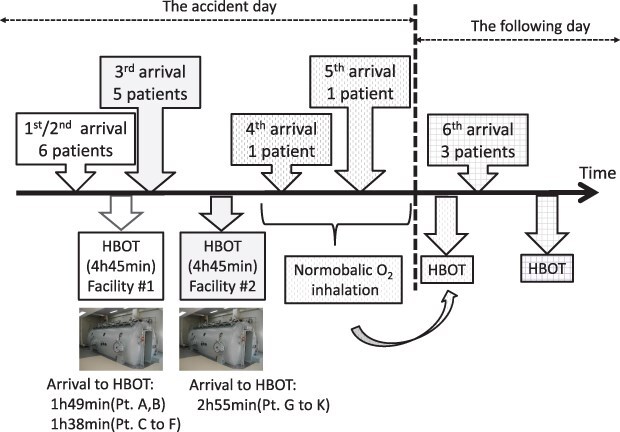
Time course of arrival to initial HBOT.

Details of the timeline are presented in **Table 2** and Figure 1. Initially, the information from the scene was confusing, and we struggled to organize a medical treatment system. However, once it became clear that at least 6 patients were involved, we opted to treat the CO exposure (and presumed ACOP) as a mass casualty. To manage the event without disrupting outpatient care, we appointed a medical control officer (MCO) and established 6 separate medical teams based in the emergency department. The MCO guided the general treatment plan and coordinated the various aspects of the plan, and the medical teams were responsible for treating the patients. The patients were triaged at the aid station on the pier, and 11 patients (A to K) were transported by ambulance to our hospital, located approximately 5 minutes away, in order of severity. Patients L and M were late-arrival walk-ins.

**Table 2.** Timeline of the events (on the day of the accident)

Time	Event	Details
∼10:30	Accident occurred	One patient with LOC on the destroyer
∼10:35	Emergency call	Nearest JMSDF MSU team (EMT, nurses) arrived at the destroyer
∼10:40		Triage at the scene by the MSU team
10:45	First call	“The accident in the destroyer may be due to ACOP.”
		“Request to accept 4 patients obtained, one with LOC.”
10:48	Mass casualty declared	Assigned an MCO, called for an HBOT team
11:00	Second call	“Request to accept 1 conscious patient.”
11:15	Third call	“Request to accept 6 patients. LOC patient recovered consciousness.”
		Assigned 6 medical teams (MD, nurses)
11:24	Patient arrival	Patients A and B, via ambulance
11:35	Patient arrival	Patients C to F, via ambulance
11:40	ACOP diagnosed	
	HBOT control (first)	The MCO approved the provision of HBOT.
11:45	Patient arrival	Patients G to K, walk-in
12:50	HBOT set-up (first)	Patients A to F transferred to our HBOT facility
13:00	HBOT control (second)	The MCO began negotiating to another HBOT facility, and assembling a second HBOT team.
13:13	HBOT start (first)	Protocol: Table 6 of the US Diving Manual ^2^
14:15	Patient arrival	Patient L, by walk-in
14:20	HBOT set-up (second)	Patients G to K, transferred to another HBOT facility
14:35	HBOT start (second)	Protocol: Table 6 of the US Navy Diving Manual ^2^
16:45	Patient arrival	Patient M, walk-in
18:10	HBOT finish (first)	No problems
19:32	HBOT finish (second)	No problems

Abbreviations: LOC, loss of consciousness; MOC, medical control officer; MSU, Medical Service Unit; EMT, emergency medical technician; HBOT, hyperbaric oxygen therapy; MD, medical doctor.

Patient characteristics are presented in **Table 1**. Upon arrival at our hospital, all patients were immediately administered normobaric oxygen via a facial mask. The results of the physical exams were unremarkable for all 16 patients, with no obvious injuries. Laboratory results, including pCO_2_ and pO_2_ levels, were within normal limits, except for carboxyhemoglobin (CO-Hb) levels, which were elevated in patients A to K.

We immediately divided the patients into 2 groups (most severe and less severe) and administered HBOT within 3 hours of the exposure to the most severely injured patients (A to F) and approximately 4 hours after the exposure to the less severely injured patients (G to K). The general conditions and vital signs of patients L and M were stable; therefore, normobaric oxygenation therapy was initially administered, and HBOT was performed the next day. The day following the accident, elevated CO-Hb levels or post-exposure symptoms despite normal CO-Hb levels were observed in outpatients N to P.

All 16 patients were diagnosed with ACOP and underwent ongoing normobaric oxygenation therapy and at least 3 HBOT sessions, as outlined in Treatment Tables (TT) 5 and 6 of the U.S. Navy Diving Manual, during hospitalization.^2^ Not all patients required myringotomy. Although symptoms resolved in all patients after the second HBOT session, 6 patients (A to F) underwent 5 sessions owing to elevated CO-Hb levels. All patients were discharged after completing their scheduled HBOT sessions and were followed up at 2 and 4 weeks after the exposure. As soon as possible after admission and at the 4-week follow-up, the patients underwent brain magnetic resonance imaging and cognitive function tests. No abnormalities were evident in any of the patients at either time.

## DISCUSSION

We report the clinical presentation and treatment of a large number of patients with ACOP occurring at an offshore location. The treatment of ACOP primarily aims to prevent (1) acute organ hypoxia, especially in the brain and myocardium, by reducing CO-Hb levels during the acute phase and (2) late-onset brain damage and neuropsychiatric disorders.^1^ Despite controversy regarding the role of HBOT in ACOP,^3–5^ some studies suggest that HBOT reduces the risk of chronic cognitive impairment in patients with specific symptoms.^6,7^ In our cohort, several criteria for ACOP were met (**Table 3**), including impaired consciousness (patients A and E), CO-Hb of 25% or more (patients A to D), and age over 36 years (patients A to F, I, J, and M to P) (**Table 3**).^1,7,8^ Although patients G, H, K, and L did not meet these criteria, they were considered eligible for HBOT because they exhibited symptoms and worked in the same confined space as did the other patients.^1^

**Table 3.** The reasons for HBOT indication

	Pt.															
Criteria	A	B	C	D	E	F	G	H	I	J	K	L	M	N	O	P
Impairment of consciousness	Yes	No	No	No	Yes	No	No	No	No	No	No	No	No	No	No	No
Initial CO-Hb > 25%	Yes	Yes	Yes	Yes	No	No	No	No	No	No	No	No	No	No	No	No
Age > 36	Yes	Yes	Yes	Yes	Yes	Yes	No	No	Yes	Yes	No	No	Yes	Yes	Yes	Yes
Existing physical symptom	Yes	Yes	Yes	Yes	Yes	Yes	Yes	Yes	Yes	Yes	Yes	Yes	Yes	Yes	Yes	No

Abbreviations: HBOT, hyperbaric oxygen therapy; Pt, patient; CO-Hb, carboxyhemoglobin.

In our study, treatment proceeded smoothly owing to the early admission of the large cohort and the appointment of an MCO to manage various adjustments. HBOT was planned, and all 16 patients received treatment as scheduled. Although a single HBOT session can last more than 4 hours,^2^ many patients can be treated simultaneously in a type-2 HBOT facility. Therefore, if the number of patients, their symptoms, and the facility’s treatment capacity and equipment performance are deemed acceptable, multiple patients can be treated simultaneously in a single facility.

We could find no consensus regarding the appropriate HBOT protocol for ACOP treatment; different protocols have been presented.^1,4^ Therefore, we performed the protocols listed in TT 5 and 6 of the U.S. Navy Diving Manual,^2^ which is frequently used at our facility. In TT 6, the patient is pressurized to 60 feet and administered oxygen for 60 minutes and then pressurized to 30 feet and administered oxygen for 120 minutes. The total treatment time is 4 hours and 45 minutes. In TT 5, the patient is pressurized to 60 feet and administered oxygen for 40 minutes and then pressurized to 30 feet and administered oxygen for 20 minutes. The total treatment time is 2 hours and 15 minutes.

We determined that patients A to F, who exhibited particularly high CO-Hb levels, should receive 5 HBOT sessions. Given their severe ACOP, we aimed to maximize oxygen administration through HBOT. However, we recognized that dispensing an excessive amount of oxygen increases the risk of oxygen toxicity. Therefore, we planned to provide an oxygen amount that would avoid this risk while still being effective. The protocol for doing so was determined under the supervision of an HBOT expert. There were no adverse effects of the treatment and no ACOP sequelae. However, the number of HBOT sessions and choice of protocol require continued discussion.

In a mass casualty event, patients should be dispersed to various facilities with appropriate treatment capabilities. Based on preliminary observations, including transient loss of consciousness in some patients, the cause of the symptoms in the present report was likely ACOP. Therefore, we determined that immediate HBOT was necessary, and 11 patients (A to K) were simultaneously admitted to our hospital. Notably, dispersing patients to more distant facilities would have required oxygenation during transport, numerous oxygen cylinders, and several ambulances, leading to a significant delay in HBOT initiation, as described by Valerio et al.^9^ In the present study, the time from ASOP onset to HBOT initiation was within 3 hours for patients A to F and approximately 4 hours for patients G to K (**Table 2**). Lee et al. reported that the neurocognitive prognosis is significantly worse when HBOT is initiated ≥6 vs. <6 hours after ACOP onset.^10^ Therefore, treating all patients at our facility was the best option, and treatment was successfully accomplished by dividing the patients into 2 severity-based groups.

Important for a successful treatment of ACOP by HBOT is a full understanding of the urgency of the situation by not only the medical staff but also receptionists, administrators, drivers, and procurers of medical equipment such as oxygen. Cooperation among these individuals and knowledge of disaster medicine by all, including the administrative staff, is essential. However, a different strategy may be required for larger exposure groups or even for the same exposure group, depending on the timing and clinical presentations. Specifically, if the number of patients exceeds the capacity of the HBOT facility, evacuation to and performance of HBOT at another facility should be considered in addition to treatment at the facility following triage. Therefore, when training HBOT personnel to handle a large number of patients, the possibility of ACOP among the patients should be taken into account.

## CONCLUSION

We initiated HBOT almost simultaneously at a single facility despite a large number of patients with ACOP and obtained good outcomes. In cases where HBOT may be beneficial and the number of nearby facilities is limited, treatment at a single facility should be considered to prioritize early treatment initiation. Assigning an MCO for local disasters and focused coordination can ensure smoother operation and effective treatment. Importantly all staff members, including managers, should understand the concept of disaster medicine, and HBOT facilities should conduct regular training sessions for potential situations involving a large number of patients with ACOP.

## Data Availability

There are no new data associated with this article.

## INSTITUTIONAL CLEARANCE

Institutional clearance does not apply.
